# Superior cervical gangliectomy induces non-exudative age-related macular degeneration in mice

**DOI:** 10.1242/dmm.031641

**Published:** 2018-02-01

**Authors:** Hernán H. Dieguez, Horacio E. Romeo, María F. González Fleitas, Marcos L. Aranda, Georgia A. Milne, Ruth E. Rosenstein, Damián Dorfman

**Affiliations:** 1 Laboratory of Retinal Neurochemistry and Experimental Ophthalmology, Department of Human Biochemistry, School of Medicine/CEFyBO, University of Buenos Aires/CONICET, Buenos Aires C1121ABG, Argentina; 2 Faculty of Medical Sciences, Pontifical Catholic University of Argentina, BIOMED/UCA/CONICET, Buenos Aires C1107AFB, Argentina

**Keywords:** Age-related macular degeneration, Superior cervical ganglion, Choroid, Retinal pigment epithelium, Photoreceptors, Experimental model

## Abstract

Non-exudative age-related macular degeneration, a prevalent cause of blindness, is a progressive and degenerative disease characterized by alterations in Bruch's membrane, retinal pigment epithelium, and photoreceptors exclusively localized in the macula. Although experimental murine models exist, the vast majority take a long time to develop retinal alterations and, in general, these alterations are ubiquitous, with many resulting from non-eye-specific genetic manipulations; additionally, most do not always reproduce the hallmarks of human age-related macular degeneration. Choroid vessels receive sympathetic innervation from the superior cervical ganglion, which, together with the parasympathetic system, regulates blood flow into the choroid. Choroid blood flow changes have been involved in age-related macular degeneration development and progression. At present, no experimental models take this factor into account. The aim of this work was to analyze the effect of superior cervical gangliectomy (also known as ganglionectomy) on the choroid, Bruch's membrane, retinal pigment epithelium and retina. Adult male C57BL/6J mice underwent unilateral superior cervical gangliectomy and a contralateral sham procedure. Although superior cervical gangliectomy induced ubiquitous choroid and choriocapillaris changes, it induced Bruch's membrane thickening, loss of retinal pigment epithelium melanin content and retinoid isomerohydrolase, the appearance of drusen-like deposits, and retinal pigment epithelium and photoreceptor atrophy, exclusively localized in the temporal side. Moreover, superior cervical gangliectomy provoked a localized increase in retinal pigment epithelium and photoreceptor apoptosis, and a decline in photoreceptor electroretinographic function. Therefore, superior cervical gangliectomy recapitulated the main features of human non-exudative age-related macular degeneration, and could become a new experimental model of dry age-related macular degeneration, and a useful platform for developing new therapies.

## Abstract (precis)

**Summary:** Ubiquitous alteration of choroid circulation causes localized retinal alterations in mice that are similar to human non-exudative age-related macular degeneration, thus providing a new potential experimental model of the disease.

## INTRODUCTION

Age-related macular degeneration (AMD) is a leading cause of irreversible blindness among people above 60 years old in industrialized countries (Jager et al., 2008; Wong et al., 2014). It is estimated to have a prevalence of ∼18% in the population aged 65-74 years (Klein et al., 1992), and the general consensus states that, as the population grows and ages, the incidence of the disease will increase (Buschini et al., 2015). AMD is a heterogeneous disease, presenting several signs such as accumulation of debris and deposits both below (drusen) and above (pseudodrusen) the retinal pigment epithelium (RPE), thickening of Bruch's membrane (BrM), hyper or hypopigmentation of the RPE, choroidal neovascularisation (CNV) and loss of photoreceptors (PRs) (Bhutto and Lutty, 2012; Jager et al., 2008). Clinically, AMD has been classified in two forms: non-exudative AMD (the more prevalent form of the disease) and exudative or neovascular AMD (NVAMD). Although the two forms might not necessarily be mutually exclusive, the hallmark of NVAMD is CNV, whereas non-exudative AMD is characterized by a delimited central area of RPE atrophy and PR loss, which in advanced stages is called geographic atrophy (GA) (Bhutto and Lutty, 2012; Jager et al., 2008; van Lookeren Campagne et al., 2014). Currently, there is no approved or effective treatment to prevent the onset and progression of GA. Several environmental aspects, such as old age, female sex, cigarette smoking, diet and Caucasian race, have been identified as risk factors for AMD (Buschini et al., 2015; Danis et al., 2015; van Lookeren Campagne et al., 2014; Zarbin, 2004). In addition, oxidative stress, inflammation, RPE senescence and choroid blood flow changes are involved in AMD development and progression (Buschini et al., 2015; Danis et al., 2015; van Lookeren Campagne et al., 2014; Zarbin, 2004). However, the exact pathogenic mechanisms and their temporal sequence are still elusive. AMD is difficult to study because of its late onset, complex genetics and the influence of environmental factors. Unravelling which are the most critical mechanisms in AMD pathogenesis is unlikely to be achieved in studies limited to the clinically observable changes in human retinas. Far more detailed and invasive studies are needed, preferably in a readily available animal model. Over the last decade, there have been an increasing number of reports describing rodent models (mostly mice), which show some characteristics compatible with human AMD (Fletcher et al., 2014; Pennesi et al., 2012; Ramkumar et al., 2010). The majority of these models result from the manipulation of mouse genes involved in inflammation (*CFH*, *CCL2*, *CCR2*, *CX3CR1*) and oxidative stress (*SOD1* and *SOD2*), as well as genes related to metabolic pathways (*CTSD*, *CP* and *APOE*), among others (Fletcher et al., 2014; Pennesi et al., 2012; Ramkumar et al., 2010). Other models include the use of a mouse strain of accelerated senescence (Majji et al., 2000) and immunization with carboxyethylpyrrole, an oxidative product of docosahexaenoic acid (Hollyfield et al., 2010). However, most of these models show AMD-like retinal changes only in old mice (12-24 months old), incorporating an established risk factor (aging), but precluding a rapid time course to allow more efficient studies, and adding an experimental difficulty because increased animal age is associated with high mortality. In addition, genetic manipulations are frequently not specific for the ocular tissues, and the retinal lesions provoked tend to be ubiquitous rather than geographic, contrary to one of the most striking findings in human dry AMD, which affects almost exclusively the macular area. Other non-exudative AMD models are induced by the systemic injection of sodium iodate, a relatively specific oxidant of the RPE (Carido et al., 2014), and a long-term exposure to high intensity light (Bordone et al., 2012). However, in these models, retinal damage occurs acutely, which is far different from the chronic and progressive changes found in human AMD. Although impairment of choroidal blood flow is one of the supposed pathogenic mechanisms of AMD (Ciulla et al., 1999; Friedman, 2008; Friedman et al., 1995), at present, there are no experimental models of the disease that specifically take into account this risk factor.

Oxygen and nutrients are supplied to the RPE and the outer retina exclusively by choroid blood flow through the BrM. Choroid blood flow is one of the highest (in volume per unit time) of the entire vascular system, providing ∼80% of the retinal blood supply, which ensures a source of oxygen and nutrients mainly to the PR layer. Choroid vessels receive sympathetic innervation from the superior cervical ganglion (SCG) (Bill, 1985; Koss, 1994; Triviño et al., 2005), which triggers vasoconstriction and, altogether with parasympathetic terminals inducing vasodilatation, regulates the flow rate through the choroid (reviewed by Nickla and Wallman, 2010). It has been shown that superior cervical gangliectomy (SCGx) induces choroid hemodynamic alterations (Steinle et al., 2002), and that a decrease in choroid blood flow correlates with BrM thickening (Pauleikhoff et al., 1990) and drusen extension (Berenberg et al., 2012). Since maintenance of an adequate choroid blood flow is essential for the proper functioning of the RPE and PRs, and that a decrease in choroid blood flow could play an important role in AMD pathogenesis, the aim of this work was to analyze the effect of SCGx on C57BL/6J mouse choroid, choriocapillaris, BrM and retina.

## RESULTS

**Fig. 1.**
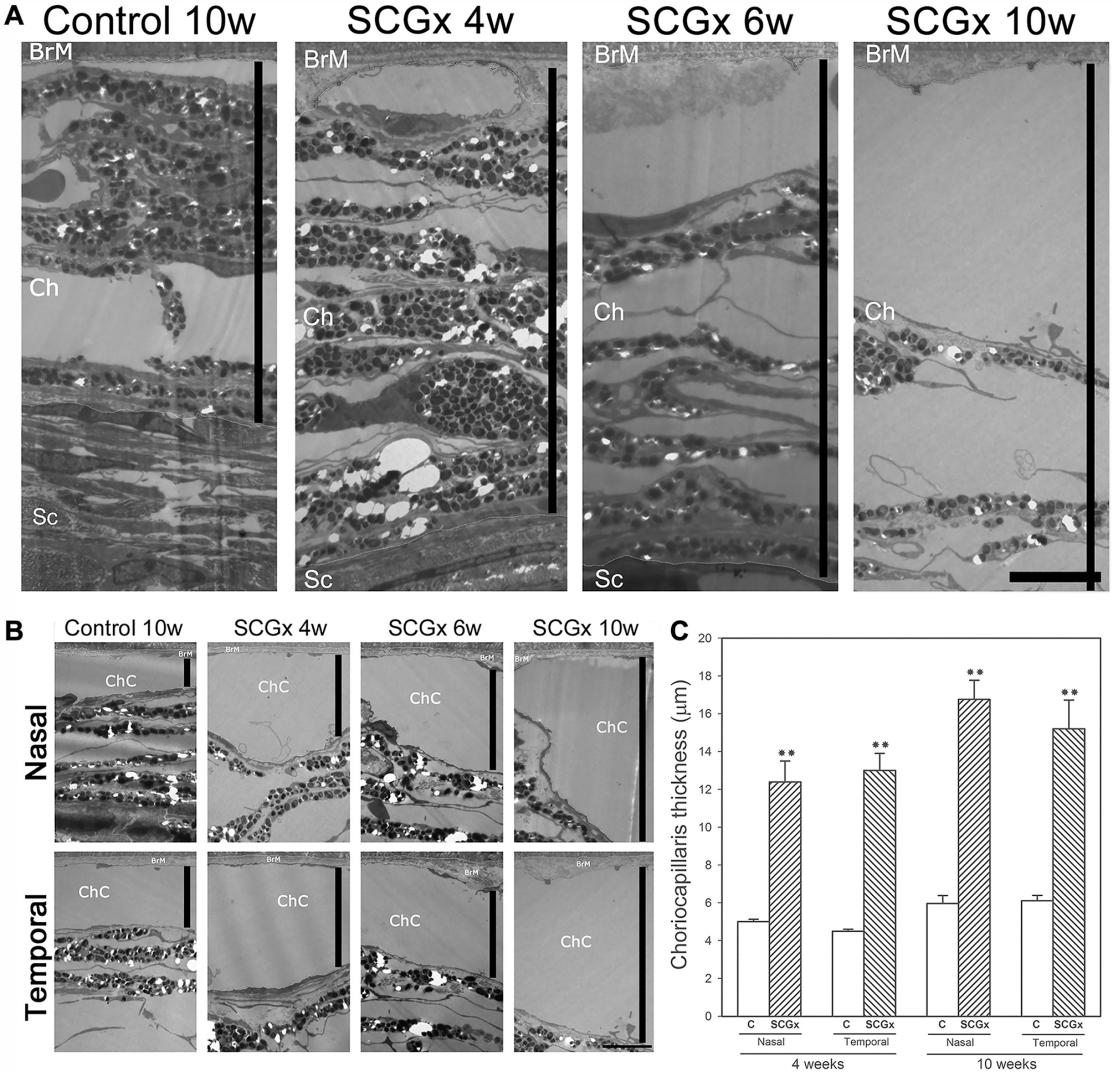
**Effect of SCGx on the choroid.** (A) Transverse ultrathin sections of the choroid from control and SCGx eyes at 4, 6 and 10 weeks post-surgery. SCGx induced an increase in choroidal thickness (black bars), tissue spreading, and vessels enlargement. (B) Choriocapillaris details from control and SCGx eyes at 4, 6 and 10 weeks post-surgery. A significant increase in nasal and temporal choriocapillaris thickness (black bars) was found in eyes submitted to SCGx at all time points. (C) Quantification of choriocapillaris thickness from control and SCGx eyes at 4, 6 and 10 weeks post-surgery. SCGx induced a significant increase in the nasal and temporal choriocapillaris thickness at 4 and 10 weeks post-surgery. Shown are representative photomicrographs from five animals/group. BrM, Bruch's membrane; ChC, choriocapillaris; Ch, choroid; Sc, sclera; C, control; SCGx, superior cervical gangliectomy. Scale bars: 5 µm. Data are means±s.e.m. (*n*=5 animals per group), **P<0.01 vs control eyes at 10 weeks post-SCGx, by Student's *t*-test.

**Fig. 2.**
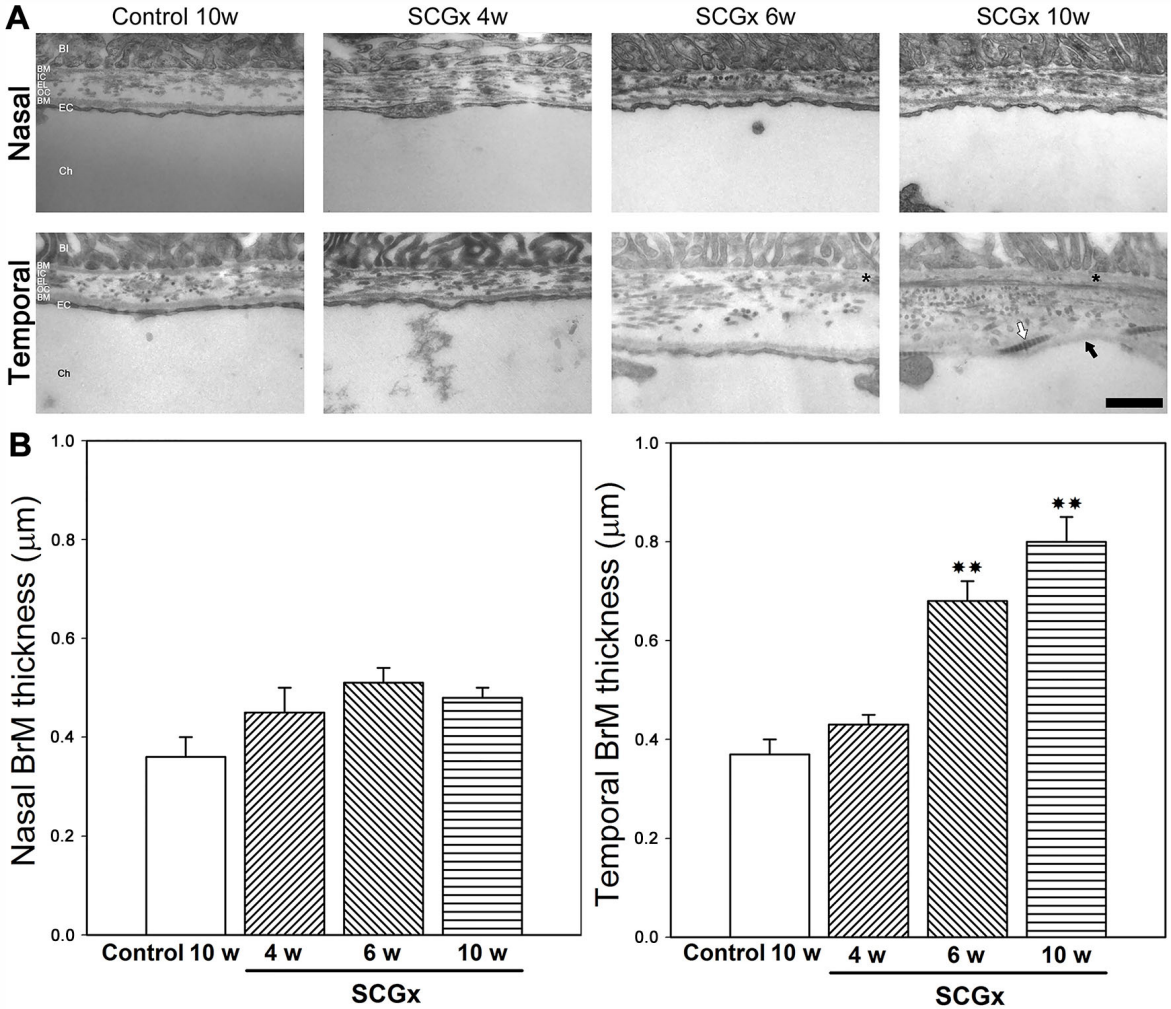
**Ultrastructural** **analysis of Bruch's membrane (BrM).** (A) Transverse ultrathin sections from control and SCGx eyes at 4, 6 and 10 weeks post-surgery. At 4 weeks post-SCGx, BrM thickness and structure were preserved, whereas, at 6 weeks post-surgery, SCGx induced a significant thickening and a clear loss of its pentalaminar structure, followed by fibrillar collagen accumulation (white arrow), reticular middle electron-dense deposits under the RPE basal membrane (asterisk) and absence of endothelial cells (black arrow) at 10 weeks post-SCGx. Shown are representative photomicrographs at 800 µm, nasally and temporally, from the ONH, from five animals/group. (B) Quantification of nasal and temporal BrM thickness in control eyes and eyes submitted to SCGx at 4, 6 and 10 weeks post-surgery. SCGx induced a significant increase in the thickness of the temporal (but not nasal) BrM after 6 and 10 weeks post-surgery. BI, RPE basal infoldings; BM, basal membrane; IC, internal collagenous layer; EL, elastic layer; OC, outer collagenous layer; EC, endothelial cell; Ch, choroid. Scale bar: 500 nm. Data are means±s.e.m. (*n*=5 animals per group), **P<0.01 vs control eyes, by Tukey's test.

**Fig. 3.**
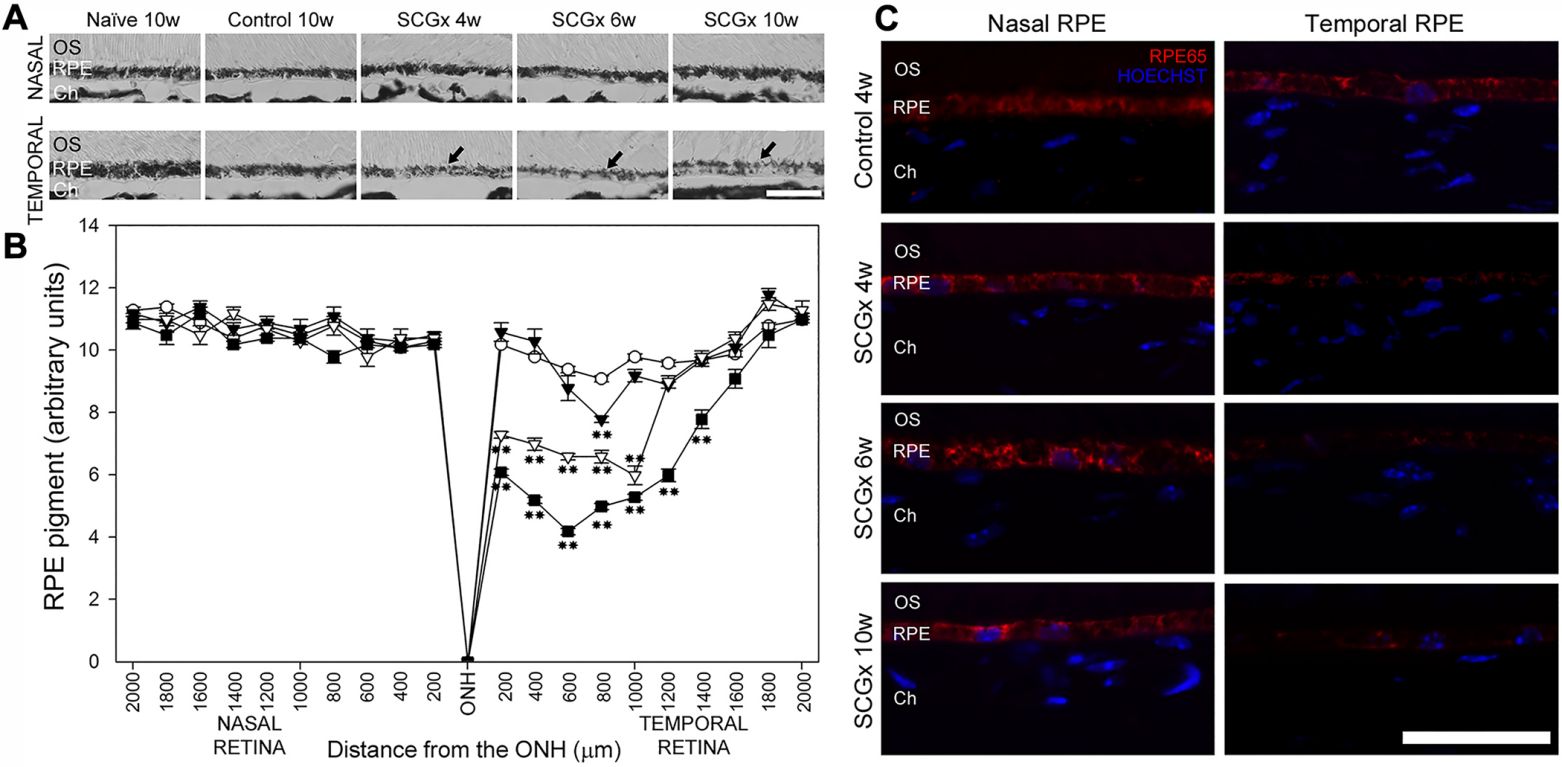
**Effect of SCGx on the RPE melanin content and RPE65 immunoreactivity.** (A) Representative photomicrographs showing RPE melanin content loss (arrows) at the temporal (but not nasal) RPE at 800 µm from the ONH. Scale bar: 25 µm. (B) SCGx induced a significant decrease in melanin content in the temporal RPE, which, at 4 weeks post-SCGx (black triangles), was evident at 800 µm temporally from the ONH and afterwards spread to almost all the temporal side, except for the periphery (1600-2000 µm from the ONH), at 6 (white triangles) and 10 (black squares) weeks post-SCGx. No differences in the nasal RPE melanin content were observed between control eyes (white circles) and SCGx eyes at all time points examined. Data are means±s.e.m. (*n*=5 animals per group), **P<0.01 vs control eyes, by Tukey's test. (C) Representative RPE photomicrographs of RPE65 immunostaining at 800 µm nasally and temporally from the ONH. A decrease in RPE65 immunostaining in the temporal (but not nasal) RPE was observed in eyes submitted to SCGx at 4, 6 and 10 weeks post-surgery. Shown are representative photomicrographs from five animals/group. OS, photoreceptor outer segments; RPE, retinal pigment epithelium; Ch, choroid. Scale bar: 50 µm.

**Fig. 4.**
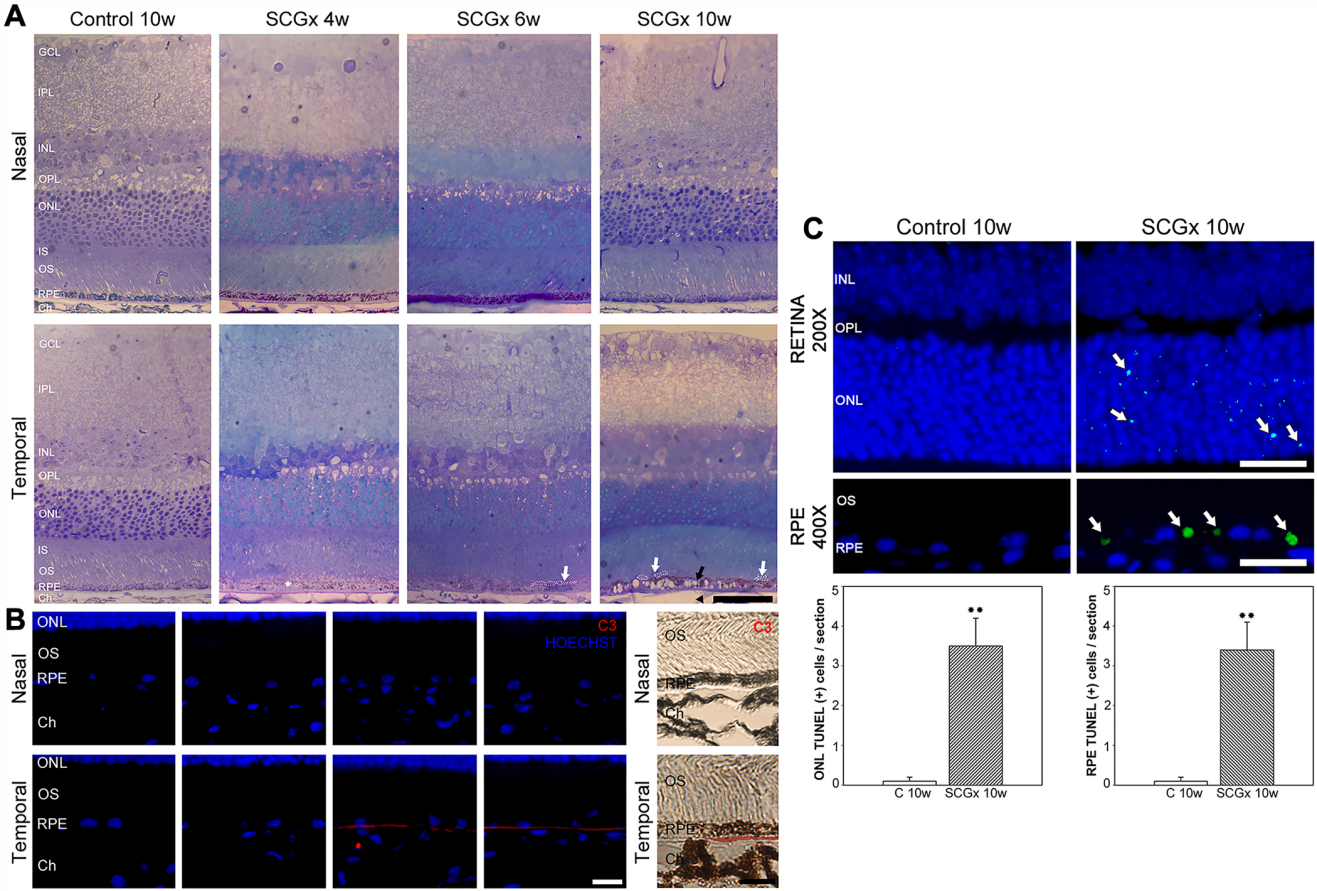
**Retinal histology, sub-retinal deposit analysis, and ONL and RPE apoptosis.** (A) Representative retinal semi-thin sections at 800 µm nasally and temporally from the ONH stained with Toluidine Blue from control eyes and eyes submitted to SCGx are shown. Besides the loss of RPE melanin content (white asterisk) at 4 weeks post-SCGx, drusenoid deposits (white arrows) between temporal RPE and PRs were found at 6 weeks post-SCGx, followed by major alterations in the temporal area such as RPE vacuolization (black arrow) and endothelial cell alterations (arrowhead), which were found at 10 weeks post-SCGx. (B) C3 immunoreactivity in representative transverse retinal sections. Linear C3(+) deposits within the temporal BrM were observed at 6 and 10 weeks post-SCGx. Shown are representative photomicrographs from five animals/group. Scale bars: 25 µm (A); 10 µm (B). (C) SCGx induced a significant increase in TUNEL(+) nuclei (arrows) in the temporal ONL and RPE at 800 µm from the ONH at 10 weeks post-surgery. Representative photomicrographs from five animals/group are shown. Scale bars: 25 μm (retina); 50 μm (RPE). RPE, retinal pigment epithelium; Ch, choroid; RPE, retinal pigment epithelium; OS, photoreceptor outer segments; INL, inner nuclear layer; IPL, inner plexiform layer; ONL, outer nuclear layer; OPL, outer plexiform layer; GCL, ganglion cell layer; IS, photoreceptor inner segments; C, control; SCGx, superior cervical gangliectomy. Data are means±s.e.m. (*n*=5 animals per group), **P<0.01 vs control eyes, by Student's *t*-test.

**Fig. 5.**
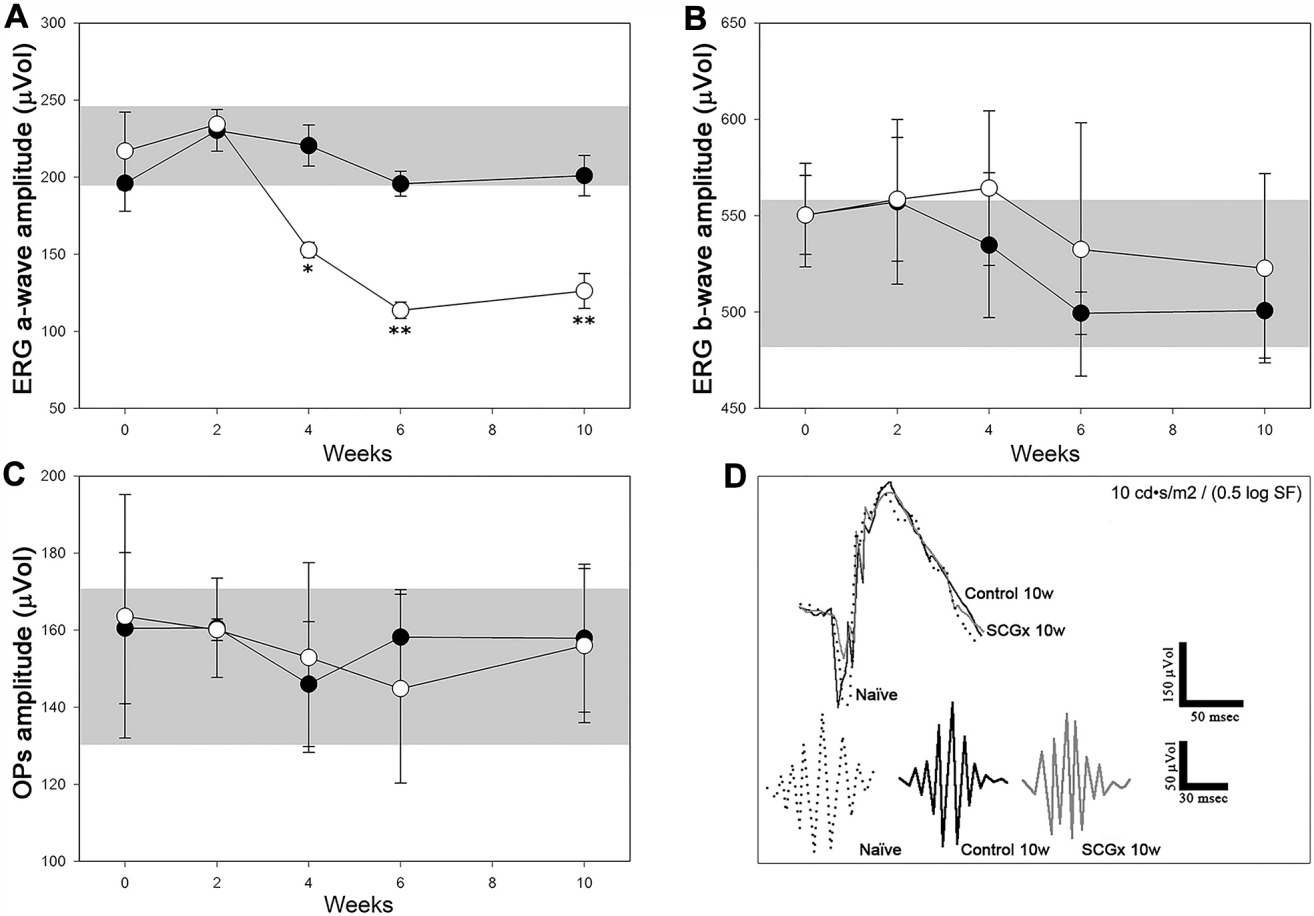
**Effect of SCGx on retinal function.** (A-C) The average amplitudes of scotopic ERG a-wave, b-wave and OPs are shown. A significant decrease in ERG a-wave amplitude was observed after 4, 6 and 10 weeks post-SCGx (white dots), whereas no alterations in the ERG b-wave and OP amplitudes were found at any time point studied. Gray bars show naïve ERG a- and b-wave and OP amplitudes. No differences were found between naïve and control eyes (black dots) in all parameters and time points studied. Data are means±s.e.m. (*n*=10 animals per group), *P<0.05 and **P<0.01 vs naïve eyes, by Tukey's test. (D) Representative scotopic ERG and OP traces of control eyes and SCGx eyes.

**Fig. 6.**
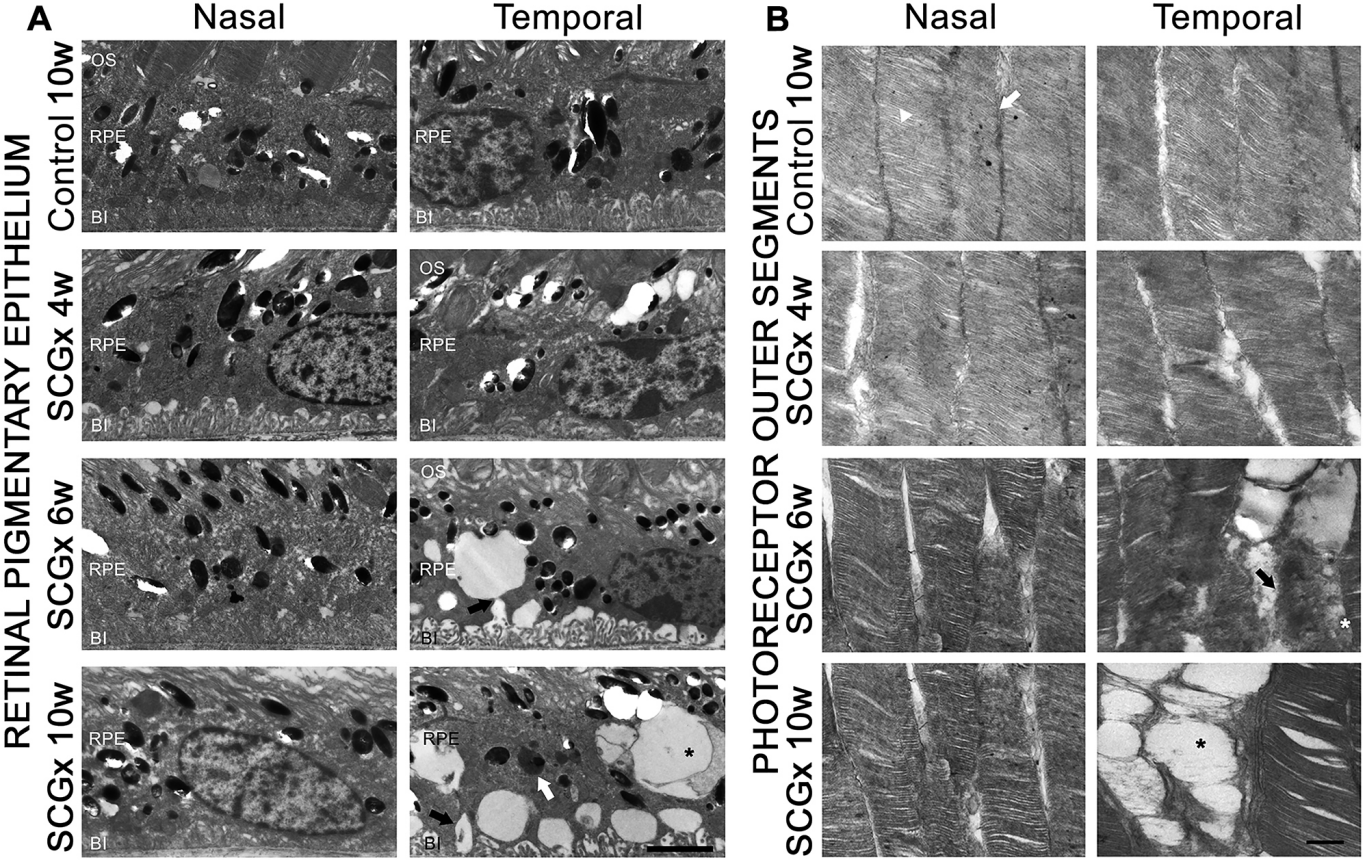
**Effect of SCGx on the RPE and PR ultrastructure.** Shown are representative transverse ultrathin RPE (A) and PR (B) sections from sham-treated eyes and eyes at 4, 6 and 10 weeks post-SCGx. (A) At 6 weeks post-surgery, SCGx induced the occurrence of vacuoles (black arrow) adjacent to the temporal (at 800 µm from the ONH) RPE basal infoldings. These changes were more evident, and vacuoles appeared more apical and often filled with membranous content (asterisk), at 10 weeks post-SCGx. Moreover, granules of middle-high electron-density were also present at the temporal RPE at 10 weeks post-SCGx (white arrow). No differences were observed at the nasal RPE between control and SCGx eyes at any time point studied. Shown are representative photomicrographs from five animals/group. OS, photoreceptors outer segments; RPE, retinal pigment epithelium; BI, basal infoldings. Scale bar: 200 nm. (B) SCGx induced focal losses of plasmatic membrane (black arrow) and the replacement of discs with an electron-dense material (white asterisk) in the temporal retina at 6 weeks post-surgery. These alterations were more evident at 10 weeks post-SCGx, and focal complete losses of discs and blebs (black asterisk) were observed. There were no alterations in nasal PR outer segment plasmatic membrane (white arrow) and discs (white arrowhead) between control and SCGx eyes at any time point studied. Shown are representative photomicrographs from five animals/group. Scale bar: 200 nm.

Representative photomicrographs of the choroid from sham-treated eyes, and eyes submitted to SCGx at 4, 6 and 10 weeks post-surgery, are shown in Fig. 1. SCGx induced an increase in choroidal thickness at 4 weeks, and this was more evident at 6 and 10 weeks post-SCGx. No differences in choroid melanin content were observed between control and SCGx eyes 10 weeks post-surgery (data not shown). A detailed analysis of the choriocapillaris showed a significant increase in its thickness both at the nasal and temporal side of the optic nerve head (ONH) at all time points studied. The sham procedure did not affect the choroid or choriocapillaris thickness (Fig. 1). No major alterations were observed among the fundus pictures of naïve, controls and eyes at 10 weeks post-SCGx (Fig. S1). SCGx induced a ubiquitous decrease in tyrosine hydroxylase (TH) immunoreactivity in the choroid, but not in the retina, at 4 and 10 weeks post-surgery. Choroid TH -immunoreactivity was similar in naïve and sham-treated eyes (Fig. S2). BrM ultrastructure was evaluated at the nasal and temporal side of the ONH at different intervals post-surgery. Alterations in BrM ultrastructure, the presence of mid electron density deposits below the RPE basal membrane and a significant increase in its thickness were observed in the temporal (but not nasal) side at 6 weeks post-SCGx, followed by an increase in fibrillar collagen content and areas with endothelial cell loss at 10 weeks post-SCGx (Fig. 2). Representative photomicrographs of the nasal and temporal RPE at 800 µm from the ONH are shown in Fig. 3. SCGx induced a significant decrease in melanin content in the temporal RPE, which, at 4 weeks after SCGx, was evident at 800 µm temporally from the ONH, and afterwards spread in a time-dependent fashion to almost all the temporal side, except for the periphery (1600-2000 µm from the ONH), at 6 and 10 weeks post-SCGx (Fig. 3A). Representative photomicrographs of RPE65 immunostaining at 800 µm from the ONH are also shown (Fig. 3C). A clear decrease in RPE65 immunoreactivity in the temporal RPE was observed at 4, 6 and 10 weeks post-SCGx (Fig. 3C). Nasal RPE melanin content and RPE65 immunoreactivity did not differ among groups at all time points studied. The effect of SCGx on the nasal and temporal retinal structure is shown in Fig. 4. Besides melanin loss, no major morphological changes in the nasal and temporal retinal structure were observed at 4 weeks post-surgery, whereas, at 6 and 10 weeks post-SCGx, drusenoid deposits were detected between RPE and PRs at 800 µm temporally from the ONH, without any obvious morphological alterations in PRs (Fig. 4). Moreover, RPE vacuolization in the temporal area was observed at 10 weeks post-SCGx. C3 immunostaining is also shown in Fig. 4. No C3 immunoreactivity was detected in sham-treated eyes or at 4 weeks after SCGx, whereas, at 6 and 10 weeks post-SCGx, the presence of C3(+) linear deposits below the temporal RPE was observed (Fig. 4). No structural alterations or C3 immunoreactivity were found in the nasal retina from all experimental groups. Fig. 4 also shows a significant increase in apoptotic cell number in the temporal outer nuclear layer (ONL) and RPE at 10 weeks post-SCGx, whereas no apoptotic cells were found in the nasal ONL and RPE, or in other retinal layers, at all time points in control retinas. There was no histological evidence of CNV, hemorrhages, serous exudates or edema in the neuroretina at any time point in any experimental group. In order to further analyze the effect of SCGx on the retina, retinal morphometry (Fig. S3), GFAP immunoreactivity and Brn3a(+) retinal ganglion cell (RGC) number (Fig. S4) were analyzed. No differences in any of these parameters were observed in the nasal and temporal retina in all experimental groups at all time points. The average amplitudes of scotopic electroretinogram (ERG) a- and b-wave and oscillatory potentials (OPs), as well as representative scotopic ERG traces, are shown in Fig. 5. In the ipsilateral retina, SCGx induced a significant decrease in ERG a-wave amplitude, which was greater at 6 and 10 weeks post-surgery (Fig. 5), whereas ERG b-wave and OP amplitudes did not change at all time points studied. In addition, no differences were observed between naïve eyes and control eyes in any of these parameters. The ERG a- and b-wave and OP latencies remained unchanged among groups (data not shown). The ultrastructural analysis showed the presence of vacuoles adjacent to RPE basal infoldings at 6 weeks post-SCGx. These changes were greater, and vacuoles appeared more apical and often filled with membranous content, at 10 weeks post-SCGx (Fig. 6). An increase in the number of granules likely containing lipofuscin and melanolipofuscin was also evident in the temporal RPE at 10 weeks post-SCGx. In the temporal retina, a clear disorganization of the membranous discs, focal loss of outer segment plasmatic membrane, and open discs were observed at 6 weeks post-SCGx (Fig. 6). At 10 weeks post-SCGx, PR blebs with complete disc loss, more evident outer segment membrane losses, and amorphous electron-dense material replacing outer segments were observed exclusively in the temporal retina. No alterations were found in control retinas along the study.

## DISCUSSION

For the first time, the foregoing results demonstrate that SCGx induces ubiquitous choroid and choriocapillaris morphological changes, as well as BrM thickening, melanin and RPE65 loss, the occurrence of drusenoid deposits, RPE atrophy, and structural alterations in PRs exclusively localized in the temporal (but not nasal) region, mimicking central features of human GA.

As expected, the sympathetic denervation induced TH immunoreactivity loss in the ipsilateral choroid, but not in the retina, from C57BL/6J mice. In addition, SCGx provoked ubiquitous distension of the choroid at 4 weeks, which persisted at 6 and 10 weeks post-surgery. An increase in choroid thickness was previously described by Steinle et al. (2002) in female Sprague-Dawley rats at 6 weeks post-SCGx that was attributed to an increase in choroidal venule number and larger choroidal arterioles. Although we did not assess these parameters in our experimental setting, the present results further support choroid thickness regulation by the SCG. Moreover, Steinle and co-workers (Steinle et al., 2002) reported that SCGx induces abnormal vascular proliferation, whereas new blood vessel formation from the choroid (CNV) that is characteristic of exudative AMD was not observed in C57BL/6J mice at least at 10 weeks post-SCGx. This inconsistency may be due to age-, gender- and species-specific differences.

A role for choriocapillaris dysfunction in AMD onset and progression has been suggested (Biesemeier et al., 2014; Nesper et al., 2017); however, to our knowledge, the choriocapillaris thickness has not been previously assessed in any other experimental model of AMD. Notably, a similar appearance of the choroid and choriocapillaris was shown in an experimental model of AMD induced by the deletion of *SOD2* in the mouse RPE (Mao et al., 2014), although, in this case, the choriocapillaris thickness was not specifically measured.

BrM lies between the choriocapillaris and the RPE, and it is a well-established ‘player’ in AMD pathogenesis (Bhutto and Lutty, 2012; Zarbin, 2004). Given its acellularity, transport across BrM primarily occurs through passive diffusion. Therefore, BrM thickening could alter its diffusion properties and, consequently, RPE and outer-retina nutrition and functioning (Bhutto and Lutty, 2012; Grindle and Marshall, 1978). The thickening of BrM has been observed in early and late stages of human AMD (Karampelas et al., 2013); however, the occurrence of BrM changes is less common in animal models of AMD (Ramkumar et al., 2010). Despite the widespread alteration in the choroid and choriocapillaris, SCGx induced BrM thickening only in the temporal (but not nasal) side at 6 and 10 weeks post-surgery. Moreover, areas with no endothelial cells adjacent to the temporal BrM were identified at 10 weeks after SCGx. In agreement, the loss of choriocapillaris endothelial cells has been proposed as a key contributor in human AMD (Chirco et al., 2017).

Eyes with non-exudative AMD are characterized by accumulation of focal extracellular-lipid- and protein-rich deposits below the RPE cells and/or within BrM, including drusen, basal laminar and basal linear deposits, which are associated with RPE dysfunction and apoptosis (Curcio et al., 2005; Hu et al., 2013; Sarks et al., 1999). Another form of retinal deposits, called reticular pseudodrusen, which, unlike conventional drusen, is located at a subretinal level, was associated with an increased risk for GA development (Fletcher et al., 2014). Small drusenoid deposits in some experimental models of AMD (Ramkumar et al., 2010; Seo et al., 2012) were mainly observed between the apical pole of the RPE and the PR outer segments (i.e. pseudodrusen), a difference that has been attributed to a simpler BrM, a different process of lipofuscin extrusion compared with humans and to the manner in which lipids are transported across the RPE in rodents (Fletcher et al., 2014; Mishima and Kondo, 1981; Ramkumar et al., 2010). An inverse correlation between choroidal blood flow and pseudodrusen area has been reported (Berenberg et al., 2012). Notably, the choroid sympathetic denervation provoked the presence of pseudodrusen deposits in the temporal (but not nasal) side. Several complement cascade components such as complement factors C3, C3b and C5 are known constituents of drusenoid deposits (Johnson et al., 2001; Mullins et al., 2000; Yao et al., 2015). In this line, sub-RPE basal laminar-like deposits containing C3 were observed at 6 and 10 weeks post-SCGx, whereas no C3 immunoreactivity was detectable in the contralateral side submitted to a sham procedure.

RPE cell dysfunction plays a central role in the subsequent PR alterations and is an important feature of AMD (Bhutto and Lutty, 2012; Jager et al., 2008; Zarbin, 2004). Melanin in the RPE absorbs light and serves as a first-line defense against PR photo-oxidation. Hyper or hypopigmentation of the macular RPE are classic findings in human (Bhutto and Lutty, 2012; Bird et al., 1995) and experimental (Rakoczy et al., 2006; Ramkumar et al., 2010) AMD. At 4 weeks post-SCGx, a loss of melanin content limited to a small area of the temporal side, which progressed to almost all (with the exception of the periphery) temporal RPE at 10 weeks post-SCGx, was observed. Damage to the RPE induced by SCGx was further supported by the loss of RPE65, an isomerohydrolase that produces 11-cis-retinol from all-trans-retinyl esters, which is specifically localized in the RPE and plays a key role in the visual cycle. Also, in this case, the decrease in RPE65 levels was observed only in the temporal RPE and already at 4 weeks after SCGx. The retinal structural damage induced by SCGx seemed to be exclusively localized in RPE and PR layer, as shown by the fact that retinal layer thickness, and GFAP (a marker for damaged Müller cells) and Brn3a (a specific marker for RGCs) immunoreactivity did not change after surgery at least at 10 weeks post-SCGx. It is well established that, in GA, RPE cells and PRs degenerate and die (Bhutto and Lutty, 2012; van Lookeren Campagne et al., 2014; Zarbin, 2004), and apoptotic PRs and RPE cells were observed in human AMD (Dunaief et al., 2002). In agreement, RPE and PR apoptotic cells were found in the temporal (but not nasal) retina from eyes submitted to SCGx.

Although not routinely used as a tool for AMD diagnosis, electroretinography can be useful to detect retinal dysfunctions arising from retinal diseases, including AMD (Justilien et al., 2007). In fact, some reports show a decreased ERG response in patients with AMD (González-García et al., 2016; Yang et al., 2016). The ERG a-wave is classically considered an index of PR activity, whereas the b-wave seems to reflect bipolar and Müller cell function, and OPs, whose origins are less clear, are thought to originate from feedback neural pathways in the inner retina, especially around the inner plexiform layer (IPL) and mainly from amacrine cells. At 4 weeks after SCGx, the ERG a-wave amplitude from the ipsilateral retina significantly decreased as compared to the contralateral retina submitted to a sham procedure, and a further decrease in this parameter was observed at 6 and 10 weeks after SCGx. In contrast, there were no differences in neither ERG b-wave nor in OP amplitudes even at 10 weeks after SCGx. There is controversy about ERG responses in experimental models of AMD, with no differences in ERG amplitudes (Vessey et al., 2015), a decrease in the ERG a-wave (Huang et al., 2017) or a decrease in both ERG a- and b-wave amplitude (Justilien et al., 2007; Mao et al., 2014; Rakoczy et al., 2006; Yao et al., 2015; Zhao et al., 2011). In those models in which a decrease in ERG response was described, functional alterations are evident in mice at age 3-17 months, whereas the effect of SCGx on retinal function was already evident at 4 weeks post-surgery. The fact that only the ERG a-wave amplitude decreased after SCGx could reflect the limited localization of the SCGx-induced retinal damage, which seems to affect exclusively the outer retina. In that sense, it should be noted that, as in other retinal injuries (Fernandez et al., 2009), ERG disturbance predated PR morphological changes, supporting that the ERG could be a sensitive indicator of retinal damage because the functional measurement revealed injury at a time when PR morphology appeared relatively normal.

Time-dependent RPE ultrastructural alterations were found in AMD human eyes (Bhutto and Lutty, 2012; Boulton and Dayhaw-Barker, 2001) and AMD-like mouse models (Mao et al., 2014; Rakoczy et al., 2006; Ramkumar et al., 2010). In the temporal RPE, progressive structural alterations, such as a decrease in melanin granules, followed by the occurrence of mid electron-dense granules (compatible with lipofuscin and/or melanolipofuscin), especially near the basal infoldings, were found and, later on, big vacuoles with membranous debris and irregular nuclei appeared. As for the PR ultrastructure, gaps between disc membranes, and even outer segment blebs and loss, were evident at 6 and 10 weeks post-surgery in the same regions in which the RPE was highly vacuolated (i.e. the temporal retina).

Two hypotheses have been proposed regarding AMD pathogenesis. One is that RPE atrophy causes choriocapillaris alterations and PR degeneration, and the other is that choroidal vascular insufficiency provokes secondary RPE and PR dysfunction and degeneration (McLeod et al., 2009). Supporting the former hypothesis, it has been demonstrated that the selective destruction of the RPE by the administration of sodium iodate (Korte et al., 1984) or mechanical debridement (Leonard et al., 2003) causes atrophy of the choriocapillaris. In contrast, it has been suggested that most cases of AMD result from alterations of the choriocapillaris in the submacular area (Duke-Elder, 1966); later on, a vascular model for AMD was proposed (Friedman, 2008) and, more recently, it has been demonstrated that choriocapillaris alterations precede RPE and retina degeneration in human AMD (Biesemeier et al., 2014). Our results demonstrate that choroid sympathetic denervation recapitulated the main features of human GA, such as focal BrM thickening, pseudodrusen, and RPE and PR alterations, supporting that a choroid/choriocapillaris alteration could be a primary event in AMD. Notwithstanding, as pointed by McLeod et al. (2009) and Bhutto and Lutty, (2012), RPE and choriocapillaris share a mutualistic relationship and, if one of the components is pathologic or compromised, either or both may become dysfunctional or may degenerate, suggesting that perhaps both hypotheses are not mutually exclusive and, consequently, in an AMD ‘scenario’ there may not necessarily be a primary insult leading to atrophy but rather a concerted deterioration affecting the entire complex (choroid/choriocapillaris/BrM/RPE/PRs).

Care must be taken when extrapolating data generated in rodents to humans. In that sense, the relevance of our study to human GA is still an open question. Genetic engineering, immunological manipulation or mouse strains with spontaneously arising retinal degeneration have been widely used to generate models that simulate some of the features of human AMD and for investigating the pathogenesis, treatment and prevention of the disease. Unlike other retinal diseases that affect the peripheral retina, AMD preferentially affects central vision and the macula. Thus, it is difficult to model this disease in the mouse because the mouse retina lacks a macula. However, it should be taken into account that recent evidence may argue on this point, since a central area with a highest rod concentration and BrM and RPE specialization were described in C57BL/6J mice (Volland et al., 2015). Why the human macula is more susceptible to degeneration in AMD is still unknown, and the same question holds for the discrete damage in the temporal region observed after SCGx in mice. Volland and co-workers (Volland et al., 2015) have shown that a central area of the mouse retina possesses some of the structural characteristics that, in the human retina, have been suggested to make the macula more susceptible to degeneration. Therefore, the identification of the mechanisms that make the temporal side more sensitive to damage induced by SCGx could contribute to understanding the particular susceptibility of the macula. In any case, even assuming that this disadvantage is shared with all other mouse models for AMD, the present results demonstrate that SCGx in C57BL/6J mice recapitulated central features of human GA, with the clear advantage of inducing AMD-like alterations in a particular region, corresponding to the mouse central retina described by Volland et al. (2015). Moreover, the relatively simple procedure of removing the SCG to induce AMD-like disease has the advantage of being applicable to larger animals with cone-rich central retinas or even to primates with macula, without needing any genetic manipulation. Furthermore, since most models in mice develop retinal lesions at older ages (6-24 months, depending on the model), the relatively early onset of AMD-like alterations induced by SCGx (i.e. at 4 weeks post-surgery) could be another advantage for the use of this model in AMD research, mainly considering that changes in the choroid/BM/RPE/retina structure and function can be influenced by aging *per se* (Curcio et al., 2000; Gao and Hollyfield, 1992; Jackson et al., 2002; Vessey et al., 2015). In this model, the progressive functional and morphological changes allow a reasonable time window to apply therapies aiming both to prevent the onset of experimental AMD (i.e. before 4 weeks post-SCGx) and to slow down the progression of the disease in different stages (i.e. between 4 and 6 weeks post-SCGx). Therefore, this new experimental model of GA could not only provide insights into AMD etiology, but help to design new therapeutic treatments for the disease.

## MATERIALS AND METHODS

### Animals

All animal use procedures were in strict accordance with the National Institutes of Health (NIH) Guide for Care and Use of Laboratory Animals. The ethics committee of the University of Buenos Aires School of Medicine [Institutional Committee for the Care and Use of Laboratory Animals (CICUAL)] approved this study. Adult male C57BL/6J mice (average weight of 27±3 g and average age of 2.5±0.5 months) were housed in a standard animal room with food and water *ad libitum*, under controlled conditions of humidity and temperature (21±2°C). The room was lighted by fluorescent lights (200 lux), which were turned on and off automatically every 12 h (on from 8.00 AM to 8.00 PM). For all experimental procedures, the animals were anesthetized with intramuscular injection of 100 mg kg^−1^ ketamine hydrochloride and 2 mg kg^−1^ xylazine hydrochloride.

### Superior cervical gangliectomy

A ventral midline incision was made in the neck and the left SCG was removed aseptically, as previously described (Romeo et al., 1991). This procedure produces complete and permanent loss of ipsilateral orbital sympathetic innervation. Care was taken to avoid carotid artery tears. Incision was closed with 7-0 nylon sutures. All mice recovered without any sign of distress. A sham procedure, without removing the right SCG, was performed, and the right eye was then considered the control eye. In some animals, while keeping the contralateral side intact, a sham procedure was performed without excision of the left SCG. In these animals, the right eye was called naïve. All animals were randomized before any experimental procedure was done and all investigators involved were blind to treatment.

### Histological evaluation

Mice were anesthetized and intracardially perfused with PBS, followed by a fixative solution containing 4% formaldehyde in 0.1 mol l^−1^ PBS (pH 7.4) using an *ad hoc* pump. Then, the eyeballs were carefully removed and post-fixed overnight in the same fixative. For orientation (nasal/temporal axis), nictitating membrane was left attached to the eye. After dehydration, eyes were embedded in paraffin wax and sectioned (5 µm) along the horizontal meridian through the ONH. Only sections where both the optic nerve and nictitating membrane were distinguishable were used. Sections were stained with hematoxylin and eosin, mounted with Canada balsam and analyzed by masked observers. Light microscopic images were digitally captured with a microscope (Eclipse E400, Nikon, Tokyo, Japan) using a 6-V halogen lamp (20 W; equipped with a stabilized light source) and a camera (Coolpix s10, Nikon, Abingdon, VA, USA). The average thickness of the total retina (TRT), IPL, inner nuclear layer (INL), outer plexiform layer (OPL), ONL and PR outer segments (OS) were measured for each eye. For each sample, measurements (×400) at 200 µm steps from the ONH to the nasal and temporal periphery were obtained and averaged from four separate sections, and the mean of five eyes was recorded as the representative value for each group.

### RPE melanin content quantification

Paraffin wax sections were deparaffinized, dehydrated and mounted in Canada balsam without any other treatment, in order to avoid any interference in the final result. Light microscopic images (×1000) were digitally captured, and analyzed by masked observers. For each eye, the total section was reconstructed and divided each 200 µm steps nasally and temporally from the ONH, taken as zero. As a result, progressively equidistant eccentricities from the ONH to the nasal or temporal periphery and a total of 20 areas, each 200 µm long, were obtained. The pigment area present only in the RPE of each area was quantified using ImageJ software version 1.42q (NIH, Bethesda, MD, USA), and the average from four separate sections per eye, and the mean of five eyes, was recorded as the representative value for each group.

### Immunohistochemical studies

Antigen retrieval was performed by heating slices at 90°C for 30 min in citrate buffer (pH 6.3). Sections were immersed in 0.1% Triton X-100 (Roche Diagnostics GmbH, Mannheim, Germany) in 0.1 mol l^−1^ PBS for 20 min for permeabilization. Sections were preincubated with 5% normal horse serum for 1 h and then were incubated overnight at 4°C with primary antibodies. The following primary antibodies were used: a rabbit polyclonal anti-TH antibody (1:500, Pel-Freeze Biologicals, Arkansas, AR, USA, P40101-0), a mouse polyclonal anti-retinoid isomerohydrolase (RPE65) antibody (1:500; EMD Millipore, Darmstadt, Germany, MAB5428), a rabbit polyclonal anti-complement component 3 (C3) antibody (1:100; Abcam, Cambridge, MA, USA, ab11887) and a mouse monoclonal anti-glial fibrillary acidic protein (GFAP) antibody conjugated to Cy3 (1:1200; Sigma Chemical Co., St Louis, MO, USA, C9205), a goat anti-Brn3a antibody (1:500; Santa Cruz Biotechnology, Inc., sc-31984). After several washings, secondary antibodies were added, and sections were incubated for 2 h at room temperature. Regularly, some sections were treated without the primary antibodies to confirm specificity. The following secondary antibodies were used: a goat anti-rabbit IgM secondary antibody conjugated to Alexa Fluor 488 (1:500; Invitrogen, Molecular Probes, Carlsbad, CA, USA, A11034), a goat anti-mouse IgM secondary antibody conjugated to Alexa Fluor 568 (1:500; Invitrogen, Molecular Probes, Carlsbad, CA, USA, A11031), a goat anti-rabbit IgM secondary antibody conjugated to Alexa Fluor 568 (1:500; Invitrogen, Molecular Probes, Carlsbad, CA, USA, A11036) and a donkey anti-goat secondary antibody conjugated to Alexa Fluor 568 (1:500; Invitrogen, Molecular Probes, Carlsbad, CA, USA, A11057). Nuclei were stained with Hoechst (1 µg ml^−1^ Sigma Chemical Co., St Louis, MO, USA), mounted with fluorescent mounting medium and observed under an epifluorescence microscope (BX-50; Olympus, Tokyo, Japan) mounted with a video camera (3CCD; Sony, Tokyo, Japan) attached to a computer running image analysis software (Optimus, Media Cybernetics, Silver Spring, MD, USA). Comparative digital images from different samples were grabbed using identical time exposition, brightness and contrast settings. Although images from the whole retinal section were taken, only images at 800 µm nasally and temporally from the ONH (as described above) are shown for practical purposes.

### Morphometric analysis

All the images obtained were assembled and processed using Adobe Photoshop SC (Adobe Systems, San Jose, CA, USA) to adjust the brightness and contrast. No other adjustments were made. For all morphometric image processing and analysis, digitalized captured TIFF images were transferred to ImageJ software version 1.42q (NIH, Bethesda, MD, USA). The analyzers were masked for treatment and time point in all experiments.

### TUNEL analysis

For DNA fragmentation of cells undergoing apoptosis, the ApopTag^®^ Fluorecein In Situ Apoptosis Detection Kit (S7110; Chemicon, CA, USA) was used according to the manufacturer's instructions. For each section, the number of TUNEL(+) cells in the ONL and RPE from the nasal or temporal retina was calculated. For each eye, results obtained from four separate sections were averaged and the mean of five eyes was recorded as the representative value for each group.

### Electron microscopy

Mice were intracardially perfused with PBS 0.1 M pH 7.4, containing 0.5 ml heparin and 2.4% sodium nitroprusside as vasodilator, followed by a fixative solution containing 2% glutaraldehyde and 4% formaldehyde. Eyes were carefully removed, and corneas and lenses removed. The nictitating membrane was left attached to the eye for orientating purposes. Eye-cups were sectioned along the horizontal meridian through the ON and post-fixed with 1% osmium tetroxide for 2 h on ice. Samples were embedded in epoxy resin. Ultrathin sections (50 nm) from the nasal and temporal retina (at 800 µm from the ONH) were obtained using glass knives and an ultramicrotome Ultracut E (Reichert-Jung, Vienna, Austria). Sections were mounted on 300 Mesh grids and stained with uranyl acetate (2% in 70% ethanol) and Reynolds lead citrate. Finally, sections were viewed and photographed using a Zeiss 109T transmission electron microscope (Carl Zeiss Microscopy, Peabody, MA, USA) equipped with a digital camera (ES1000W; Gatan, Pleasanton, CA, USA).

### ERG recording

Standard scotopic electroretinographic activity was assessed before and at 2, 4, 6 and 10 weeks post-unilateral SCGx. After an overnight dark adaptation, mice were anesthetized under dim red illumination. Phenylephrine hydrochloride and tropicamide were used to dilate the pupils, and the cornea was intermittently irrigated with balanced salt solution to maintain the baseline recording and to prevent keratopathy. Recordings were made with a HMsERG model 2000 (Ocuscience LLC, Kansas City, MO, USA) equipped with a Ganzfield dome fitted with a white-light-emitting-diode stimulus at a distance of 2 cm from the eye. For each test, 15 full-field flashes (2 ms each) separated by a 60 s interval [flash intensity 10 cd s m^−2^ (candela seconds per square meter) or 0.5 log standard flash (SF)] were averaged. The band pass of the amplifiers was 0.3-300 Hz. A reference electrode was placed through the ear, a grounding electrode was attached to the tail and a silver embedded thread electrode with a 2.5 mm lens (Ocuscience, Rolla, MO, USA) was placed in contact with the central cornea. A 15 W red light was used to enable accurate electrode placement. This procedure did not significantly affect dark adaptation and was switched off during the electrophysiological recordings. The a-wave was measured as the difference in amplitude between the recording at onset and the trough of the negative deflection, and the b-wave amplitude was measured from the trough of the a-wave to the peak of the ERG. Mean values from each eye were averaged, and the resultant mean value was used to compute the group means a- and b-wave amplitude±s.e.m. OPs were assessed by filtering of the ERG recordings applying filters of high (140 Hz) or low (100 Hz) frequency with HMsERG software version 3.6 (Ocuscience, Rolla, MO, USA). The amplitudes of the OPs were estimated by measuring the heights from the baseline drawn between the troughs of successive wavelets to their peaks. Mean values from each eye were averaged, and the resultant mean value was used to compute the group OPs amplitude±s.e.m.

### Fundus imaging

After 10 weeks post-SCGx, mice were anesthetized as described above, pupils were dilated with 2.5% phenylephrine, and 0.5% proparacaine (Alcon Laboratories, Argentina) was applied for topical anesthesia. When full mydriasis was achieved, the anesthetized animal was placed in lateral recumbency under the surgical microscope (LAB 5 LED, Newton, Buenos Aires, Argentina) with coaxial light, and positioned with one holding hand. The fundus was visualized with the application of a slide glass, with a drop of 2.5% methylcellulose (Poen Laboratories, Argentina) placed on the contact area of the cornea. Fundus photographs from the central retina (i.e. when the optic nerve head appeared in the center of the screen) were obtained using a digital camera (Coolpix s10; Nikon, Abingdon, VA, USA) adapted to the microscope.

### Statistical analysis

Statistical analysis of results was made by a Student's *t*-test or a one-way analysis of variance (ANOVA) followed by a Tukey's test, as stated, and met the necessary assumptions. The assumption of equal variances was tested by the *F*-test. In every statistical analysis, *P*<0.05 was considered statistically significant.

## Supplementary Material

Supplementary information
