# Supplementary material for: Superior cervical gangliectomy induces non-exudative age-related macular degeneration in mice
Source: Dis Model Mech. 2018 Feb 1;11(2):dmm031641. doi: 10.1242/dmm.031641 (PMC5894943; doi:10.1242/dmm.031641)
Supplement: Supplementary information [file dmm-11-031641-s1.pdf]

SUPPLEMENTARY MATERIAL

Figure S1

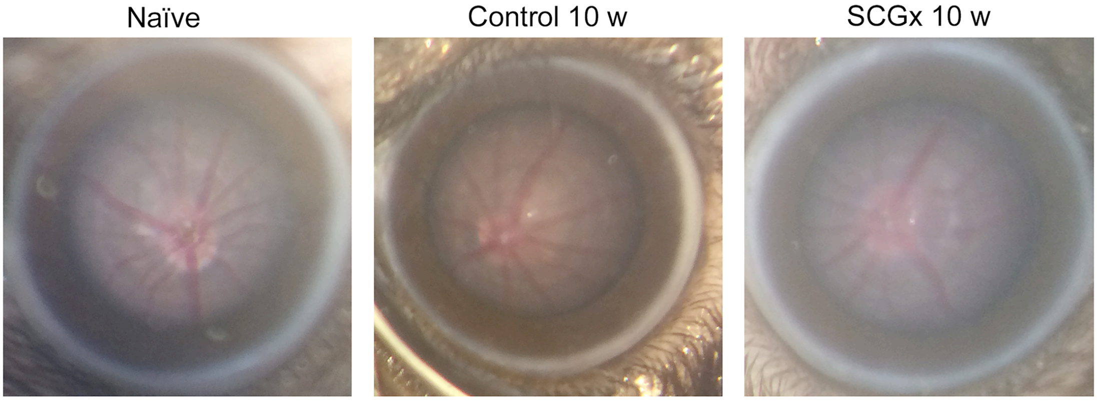

Figure S1. Fundus images analysis at 10 weeks post-SCGx. There were no major differences regarding vascular distribution, optic nerve head, and pigmentation in the temporal or nasal fundus among naïve, sham- and SCGx-treated eyes at 10 weeks post-surgery. Shown are photographs representative from 5 animals per group.

Figure S2

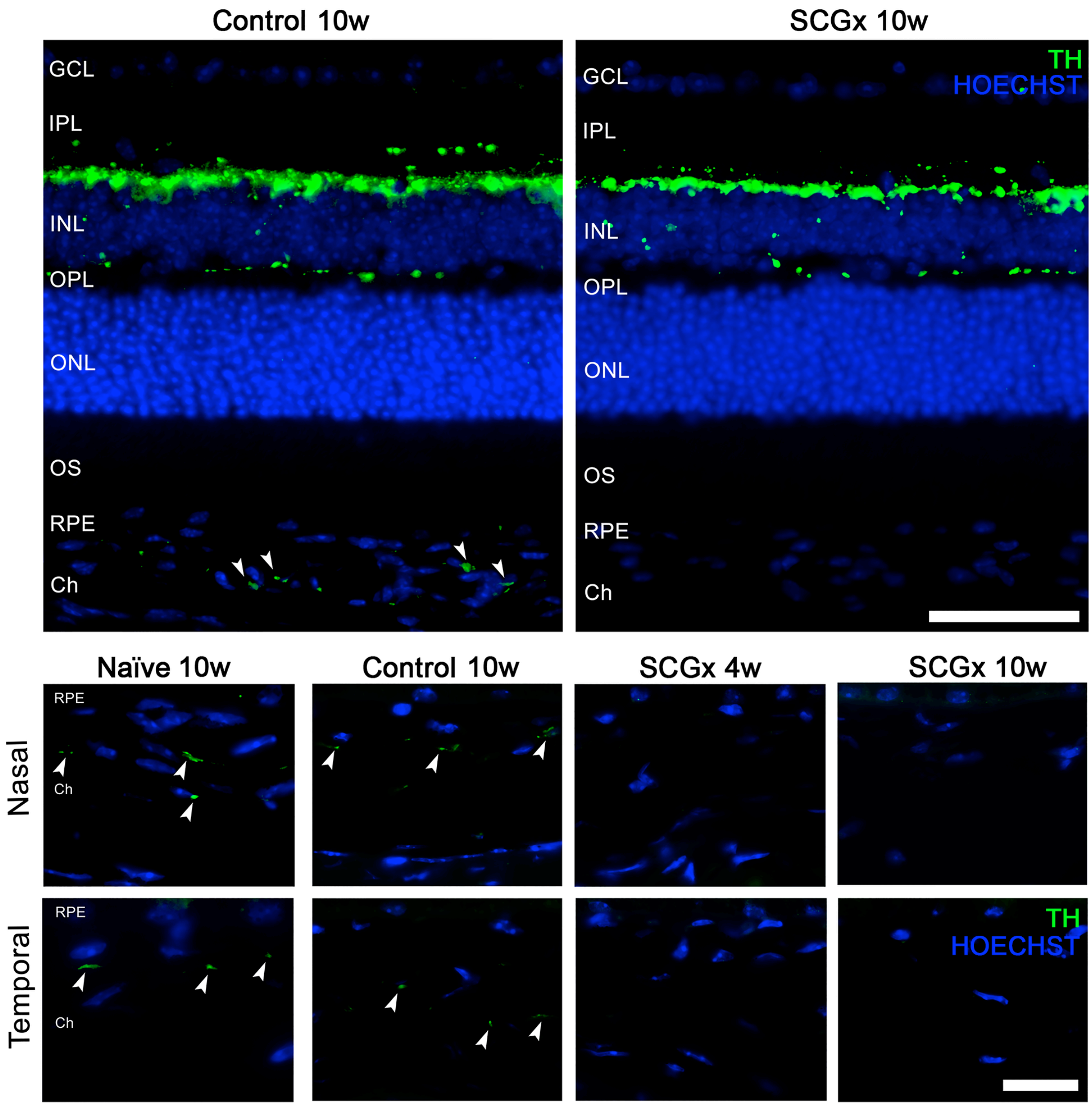

Figure S2. Effect of SCGx on TH-immunoreactivity. Upper panel: TH-immunoreactivity was observed in the IPL and choroid (arrowheads) in control eyes. At 10 weeks post-SCGx, retinal TH-immunoreactivity remained unchanged, whereas choroidal TH-immunostaining was undetectable. Lower panel: Magnified detail of the choroid from naïve, control, and SCGx-eyes at 4 and 10 weeks post-surgery are shown. Shown are images representative of 5 animals/group. Ch, choroid; RPE, retinal pigment epithelium; OS, photoreceptor outer segments; INL, inner nuclear layer; IPL, inner plexiform layer; ONL, outer nuclear layer; OPL, outer plexiform layer; GCL, ganglion cell layer. Scale bar upper panel = 50 μm; lower panel = 10 μm.

Disease Models & Mechanisms • Supplementary information

Figure S3

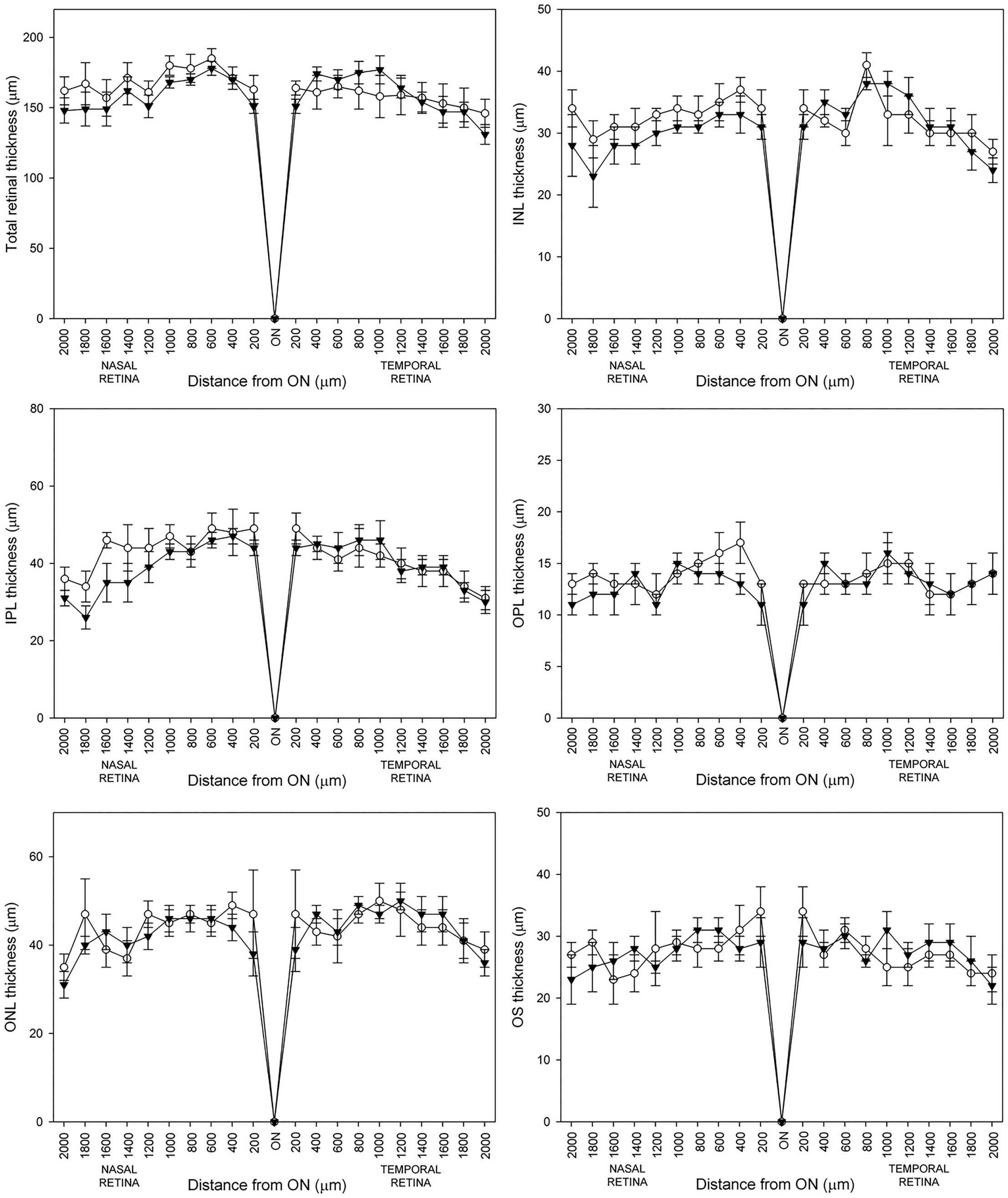

Figure S3. Effect of SCGx on retinal morphometry. There were no significant differences between control- and SCGx-eyes at 10 weeks post-surgery at any eccentricity in any parameter studied. TRT, total retinal thickness; IPL, inner plexiform layer; INL, inner nuclear layer; OPL, outer plexiform layer; ONL, outer nuclear layer; OS, photoreceptor outer segments. Data are mean ± s.e.m. (n: 5 eyes per group), by Student´s t-test.

Figure S4

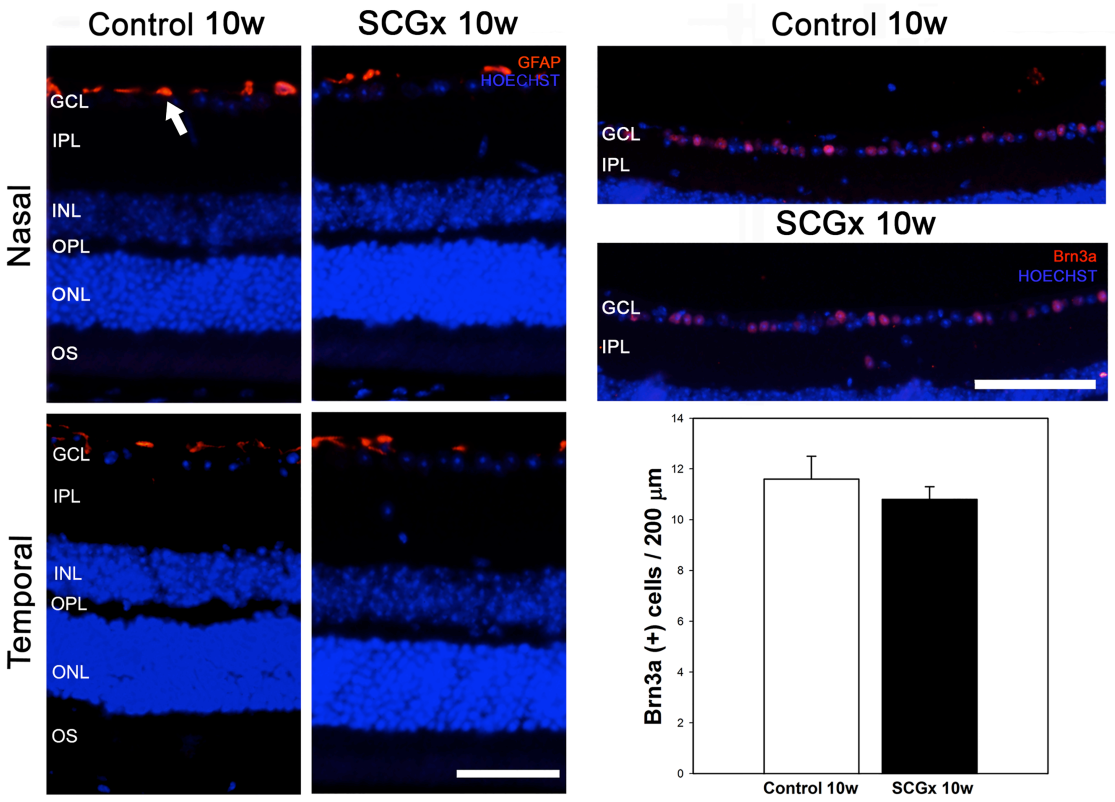

Figure S4. Effect of SCGx on RGC number and glial reactivity. Left panel: GFAP-immunoreactivity was confined to the astrocytes surrounding RGC layer (arrow) in all experimental groups. SCGx did not affect GFAP-immunostaining in Müller cell processes at 10 weeks post-surgery. Right panel: No significant difference in Brn3a(+) cell number was observed between control and SCGx-eyes at 10 weeks post-surgery. GCL, ganglion cell layer; IPL, inner plexiform layer; INL, inner nuclear layer; OPL, outer plexiform layer; ONL, outer nuclear layer, OS, photoreceptor outer segments. Representative photomicrographs of 5 animals per group are shown. Scale bar left panel = 50 µm; right panel = 100 µm. Data are mean ± s.e.m. (n: 5 eyes per group), by Student´s t-test.
